# Supplementary material for: A Critical View of the Application of the APEX Software (Aqueous Photochemistry of Environmentally-Occurring Xenobiotics) to Predict Photoreaction Kinetics in Surface Freshwaters
Source: Molecules. 2019 Dec 18;25(1):9. doi: 10.3390/molecules25010009 (PMC7017383; doi:10.3390/molecules25010009)
Supplement: Supplementary file 1 [file molecules-25-00009-s001.zip › 3.2.4_gcc-4.4.0.zip!/3.2.4_gcc-4.4.0/3.2.4_gcc-4.4.0/doc/octave/html/interpreter/Emacs-Octave-Support.html]

Emacs Octave Support - Untitled


Next: Copying,
Previous: Installation,
Up: Top


---

## Appendix G Emacs Octave Support

The development of Octave code can greatly be facilitated using Emacs
with Octave mode, a major mode for editing Octave files which can e.g.
automatically indent the code, do some of the typing (with Abbrev mode)
and show keywords, comments, strings, etc. in different faces (with
Font-lock mode on devices that support it).

It is also possible to run Octave from within Emacs, either by directly
entering commands at the prompt in a buffer in Inferior Octave mode, or
by interacting with Octave from within a file with Octave code. This is
useful in particular for debugging Octave code.

Finally, you can convince Octave to use the Emacs info reader for
`help -i`.

All functionality is provided by the Emacs Lisp package EOS (for “Emacs
Octave Support”). This chapter describes how to set up and use this
package.

Please contact <Kurt.Hornik@wu-wien.ac.at> if you have any questions
or suggestions on using EOS.

- Installing EOS- Using Octave Mode- Running Octave From Within Emacs- Using the Emacs Info Reader for Octave
